# A feature selection method based on multiple kernel learning with expression profiles of different types

**DOI:** 10.1186/s13040-017-0124-x

**Published:** 2017-02-02

**Authors:** Wei Du, Zhongbo Cao, Tianci Song, Ying Li, Yanchun Liang

**Affiliations:** 1 College of Computer Science and Technology, Key Laboratory of Symbol Computation and Knowledge Engineering of the Ministry of Education, Jilin University, Changchun, 130012 China; 2 Zhuhai Laboratory of Key Laboratory of Symbolic Computation and Knowledge Engineering of Ministry of Education, Zhuhai College of Jilin University, Zhuhai, 519041 China; 3 School of Management Science and Information Engineering, Jilin University of Finance and Economics, Changchun, 130012 China

## Abstract

**Background:**

With the development of high-throughput technology, the researchers can acquire large number of expression data with different types from several public databases. Because most of these data have small number of samples and hundreds or thousands features, how to extract informative features from expression data effectively and robustly using feature selection technique is challenging and crucial. So far, a mass of many feature selection approaches have been proposed and applied to analyse expression data of different types. However, most of these methods only are limited to measure the performances on one single type of expression data by accuracy or error rate of classification.

**Results:**

In this article, we propose a hybrid feature selection method based on Multiple Kernel Learning (MKL) and evaluate the performance on expression datasets of different types. Firstly, the relevance between features and classifying samples is measured by using the optimizing function of MKL. In this step, an iterative gradient descent process is used to perform the optimization both on the parameters of Support Vector Machine (SVM) and kernel confidence. Then, a set of relevant features is selected by sorting the optimizing function of each feature. Furthermore, we apply an embedded scheme of forward selection to detect the compact feature subsets from the relevant feature set.

**Conclusions:**

We not only compare the classification accuracy with other methods, but also compare the stability, similarity and consistency of different algorithms. The proposed method has a satisfactory capability of feature selection for analysing expression datasets of different types using different performance measurements.

**Electronic supplementary material:**

The online version of this article (doi:10.1186/s13040-017-0124-x) contains supplementary material, which is available to authorized users.

## Background

With the development of transcriptomics research, especially the widely used high-throughput microarray chip and RNA sequencing technology, a large number of transcriptome data have been obtained by measuring the expressions of genes or miRNAs simultaneously. Researchers can acquire these different expression data from several public databases, such as Gene Expression Omnibus (GEO) [1], Stanford Microarray Database (SMD) [2], ArrayExpress [3] and The Cancer Genome Atlas (TCGA) [4]. TCGA is the largest cancer genome sequencing project, which plan to sequence and organize 10,000 cancer genomes, along with other matching omics data types, covering 25 cancer types [5]. Developing effective and robust methods to extract the subset of informative features from expression data remains a challenge and crucial problem. Feature selection technology has been studied and applied proverbially in pattern recognition, statistics analysis, data mining and machine learning [6]. In the last decade, feature selection technology has become an important tool for expression data analysis in the field of bioinformatics, such as cancer classification, biological network inference, expression correlation analysis and disease biomarker identification [7]. The features (mRNAs or miRNAs) of given expression data can be broadly categorized into three major types: relevant features, redundant features and irrelevant features [8].

In general, most feature selection methods can be divided into three categories: filter methods, wrapper methods, and embedded methods [7]. These categories depend on the combination modality of feature selection search and the construction of the classification model. **Filtering methods**, which are independent of the classifier, select relevant features only dependent the intrinsic properties of expression data. Glaab et al. applied an ensemble filter method which combines several selection schemes to an ensemble feature ranking [9]. Cai et al. proposed a feature weighting algorithm to estimate the feature weights through local approximation rather than global measurement. Experimental results on both synthetic and real microarray datasets validated that the algorithm was effective, when combining the proposed method with classic classifiers [10]. Cao et al. proposed a filtering feature selection method for paired microarray expression data analysis [11].

In **wrapper approaches**, the classification scores for features by a classifier are measured in the selection process and the step of feature selection depends on the classifier. So far, many wrapper feature selection methods have been proposed and used for expression data analysis. Mukhopadhyay et al. combined a multi-objective genetic algorithm and SVM classifier as a wrapper for evaluating the chromosomes that encode miRNA feature subsets [12]. Maulik et al. presented a fuzzy preference based rough set method for feature selection from gene expression data of microarray. Compared with signal-to-noise ratio and consistency based Feature Selection methods, experimental results showed that the method was effective in extracting gene markers [13].

In **embedded approaches**, the step of selecting an optimal feature subset is built into the classifier construction and the selecting can be seen the process combined space of feature subsets and hypotheses. With the increase of available expression data sources, several embedded feature selection methods have been presented to analyze expression data. Chen et al. proposed a feature selection approach using the information provided by the separating hyperplane and support vectors [14]. Mao et al. proposed a unified feature selection framework based on a generalized sparse regularizer for measuring the performance of multivariate [15]. Li et al. proposed a new feature selection algorithm called feature weighting as regularized energy-based learning. The experiments using microarray data demonstrated that the ensemble method, when using the L2 regularizer outperforms other algorithms in stability while providing comparable classification accuracy [16]. Kursa compared four state-of-the-art Random Forest-based feature selection methods in the gene selection context on microarray datasets, and found when the number of consistently selected genes was considered, the Boruta algorithm was the best one [17]. Yousef et al. developed a method for selecting significant genes, which uses K-means to identify correlated gene clusters and applies the scores of those gene clusters for the purpose of classification [18]. Tang et al. presented a two-stage Recursive Feature Extraction (RFE) algorithm, which can effectively eliminate most of the irrelevant, redundant and noisy genes, and select informative genes in different stages [8]. Niijima et al. suggested a recursive feature elimination model based on Laplacian linear discriminant analysis for feature selection [19]. However, these methods based on RFE may obtain satisfactory performance on hundreds of features. Such a large number of features (mRNAs or miRNAs) are difficult to apply to several fields, such as clinical diagnosis of cancer or experiments of identifying cancer biomarkers.

In recent years, several **hybrid feature selection approaches** have been also proposed for expression data analysis. Chuang et al. proposed a feature selection method, which combines an improved particle swarm optimization with the K-nearest neighbor method and support vector machine classifiers [20]. Mundra et al. developed a hybrid feature selection method by combining the filter method of minimum-redundancy maximum-relevancy (MRMR) and the wrapper method of support vector machine recursive feature elimination (SVM-RFE) [21]. Du et al. proposed a multi-stage feature selection method for microarray expression data analysis [22].

Though most of above methods can eliminate the irrelevant genes and rank informative genes effectively, they are only suitable for expression data from one type of expression profile. Most of the above methods construct the feature selection model based on one type of expression data directly, but they rarely consider the effectiveness and stability on expression data from different types of transcriptome. In this paper, we propose a novel two-stage feature selection method which uses multiple kernel learning (MKL) [23, 24] combines a forward feature selection procedure to select the relevant feature subset, eliminate redundant features and select compact feature subsets. We simplify our proposed method as Simple MKL-Feature Selection (SMKL-FS), which eliminates irrelevant features and selects relevant features by the score of individual feature, and eliminates redundant features by the forward selection procedure in two stages.

One objective of feature selection is to avoid overfitting and improve the performance of classifier [7]. Overfitting is one of challenging problems on gene expression data which have characteristic of high dimensional and small sample. So, we used following processing to decrease the influence of overfitting on small samples. Firstly, we use the SimpleMKL method, which solves the MKL problem through a primal formulation involving a weighted l2-norm regularization. The regularization part adds a cost term for bringing in more features with the objective function. Hence, regularization can shrink the coefficients of many variables to zero and decrease the overfitting. Secondly, we used a sequential forward selection (SFS) method which belonged to deterministic methods and have lower overfitting risk than randomized methods [7]. In addition, we used cross validation in performance measurement part to identify these methods, which may have poor performance caused by overfitting training on several datasets.

In the following part, we outline the main steps of SMKL-FS. Firstly, we measure the relevance between features and classify samples by using the optimizing function of MKL. More specifically, we use an iterative gradient descent process to perform the optimization both on the parameters of SVM and kernel confidence, and obtain the optimizing function of each feature. Then, we select the relevant features set by sorting the optimizing function of each feature. Furthermore, we apply an embedded scheme of forward selection to detect the compact feature subsets from the relevant features set. Different from wrapper approaches, which convolve with a classifier and minimize the classification error of the dependent classifiers, we use optimizing function of MKL instead of classification error to carry out the embedded process. The idea of this process is similar as the minimum-redundancy process in mRMR [25]. Except for evaluating the classification accuracy of the method, we measure the performances of different feature selection algorithms through measuring the stability of feature space on different samples in the same type of data, the similarity with other methods and consistency between expression data of miRNA and mRNA.

The main characteristics of our proposed algorithm include: (i) a novel feature selection method for identifying gene signatures based on multiple kernel learning focusing on multiple types of expression data, such as mRNA microarray, mRNA sequencing and miRNA sequencing; (ii) an evaluattion performance of different methods by using classification accuracy, stability of feature space, similarity with other methods and consistency between expression data of miRNA and mRNA. Experimental results show that the proposed method has a satisfactory capability of feature selection for different expression datasets analysis compared to other state of art feature selection approaches.

## Results

For measuring the performance of embedded method, we use three kernel functions, linear kernel *K*(*x*_*i*_, *x*) = (*x*_*i*_, *x*), radial basis function kernel $K\left({x}_i, x\right)= \exp \left(-\frac{{\left\Vert {x}_i- x\right\Vert}^2}{2}\right)$ and polynomial kernel *K*(*x*_*i*_, *x*) = [(*x*_*i*_, *x*) + 1]^2^. In a practical application, different kernels can combined. The features are selected and evaluated using 10-fold Cross-Validation (CV) on a variety of datasets through different feature selection methods including SVM-RFE [26], SVM-RCE [18], mRMR [25], IMRelief [10], SlimPLS [27] and SMKL-FS. We measure the performances of different feature selection algorithms through evaluating the classification accuracy of feature combination, also measuring the stability of feature space on different samples in the same type of data and the similarity with other methods.

### Data sources and pre-processing

In this paper, three types of expression data are used to measure the performance of feature selection methods. We only use the paired samples in expression datasets which include tumor and adjacent non-tumor tissues. The datasets of mRNA microarray are obtained from Gene Expression Omnibus (GEO) [1], the datasets of mRNA sequencing and miRNA sequencing are downloaded from The Cancer Genome Atlas (TCGA) [4]. Eight types of cancer on microarray datasets are used in this article, and each type of cancer contains several datasets (series in GEO). Table 1 gives the more detailed information of the eight cancer types of mRNA microarray datasets from GEO and Table 2 shows the more detailed information of the eight cancer types from TCGA.

**Table 1:** The detailed information of mRNA microarray datasets

Cancer Type	Datasets ID	Number of Samples
Liver	GSE5364, GSE22058, GSE14520, GSE12941	132
Pancreatic	GSE15471, GSE16515, GSE22780	63
Lung	GSE5364, GSE19804, GSE22058, GSE10072, GSE7670, GSE2514	249
Colon	GSE5364, GSE8671, GSE25070, GSE21510, GSE23878, GSE18105	70
Gastric	GSE13911, GSE13195, GSE5081, GSE19826	93
Breast	GSE5364, GSE15852, GSE10810, GSE16873, GSE5764, GSE14548	113
Thyroid	GSE5364, GSE3678	23
Prostate	GSE6919, GSE6956, GSE17951	88

**Table 2:** The detailed information of mRNA Sequencing and miRNA Sequencing datasets

Cancer Type	Number of Samples
KIDNEY^1^	88
BRCA	71
LUNG^2^	47
HNSC	37
LIHC	46
PRAD	43
STAD	29
THCA	56

1: KIDNEY contains KIRC and KIRP

2: LUNG contains LUSC and LUAD

For using these expression data to measure the performance of different feature selection methods, the downloaded and reorganized data from GEO and TCGA have been converted in our defined data format and preprocessed through the following processes. Firstly, the missing values of each expression dataset are estimated. If the missing values of one mRNA (or miRNA) are less than 20% of all samples, these missing values are estimated using the local least squares imputation (LLSimpute) method [28]. Then, the different probes of the same mRNA (or miRNA) are merged by the maximum expression value of these probes for each sample. After these processes, these datasets are normalized by median absolute deviation (MAD) method to make all the samples have similar background [29]. The normalization of different microarrays is applied by housekeeping gene as performed in previous article [30].

### Performance measurement of feature space

The performance measurement of feature space is important for evaluating different feature selection algorithms. Most of the state of art algorithms only validate their performance through the classification accuracy [26] or classification error [31] on selected feature set by a classifier *C*. The classification accuracy and classification error are defined as follows respectively:

1 $$ \begin{array}{l}\mathrm{Accuracy}=\frac{TP+ TN}{FN+ TP+ TN+ FP}\hfill \\ {}\mathrm{Classification}\kern0.5em \mathrm{Error}=\frac{FN+ FP}{FN+ TP+ TN+ FP}\hfill \end{array} $$

where *TP* is the number of true positives, *TN* is the number of true negatives, *FP* is the number of false positives, and *FN* is the number of false negatives. However, only computing the classified ability of selected features could not reflect the performance of feature selection algorithms roundly.

In this paper, we measure the performances of different feature selection algorithms through evaluating the classification accuracy of single features and features combination, also measuring the stability of feature space on different samples in the same type of data, the similarity with other methods and consistency between expression data of miRNA and mRNA. We select and evaluated features using 10-fold Cross-Validation (CV) on these datasets mentioned above through different feature selection methods, SVM-RFE [26], SVM-RCE [18], mRMR [25], IMRelief [10], SlimPLS [27], OSFS [32], FGM [33] and our method SMKL-FS. Firstly, for each testing dataset, we randomly selected 90% as training dataset and other 10% as test dataset. Repeating the selection process 10 times, we can obtain a collection of 10 groups contained training and test samples. In order to ensure fairness, we select feature subset using each feature selection method on training samples of the same 10 groups. Then, for the ten selected features from different methods, we evaluate them according to the above criterions.

#### Classification accuracy of features combination

For two feature sets ${S}_{{}_n}^1$ and ${S}_{{}_n}^2$, and the above classifier *C*, we consider the feature space of ${S}_{{}_n}^1$ is more *effective*, if the classification accuracy on feature set ${S}_{{}_n}^1$ is higher than that on ${S}_{{}_n}^2$ by using classifier *C*. If the method *M* ^1^ generates a series of feature subsets in ${S}_{{}_n}^1:{S}_1^1\subset {S}_2^1\subset \dots {S}_{n-1}^1\subset {S}_n^1$ and the method *M* ^2^ generates a series of feature subsets in ${S}_{{}_n}^2:{S}_1^2\subset {S}_2^2\subset \dots {S}_{n-1}^2\subset {S}_n^2$. For each *k*(1 ≤ *k* ≤ *n*), we compute the classification accuracy on *S*_*k*_^1^ and *S*_*k*_^2^ as same as [8]. If the average of these classification accuracies on ${S}_{{}_n}^1$ is higher than that on ${S}_{{}_n}^2$, we consider the method *M* ^1^ is better than *M* ^2^ in ***mean effectiveness***. If the maximum of these classification accuracies on ${S}_{{}_n}^1$ is higher than that on ${S}_{{}_n}^2$, we consider the method *M* ^1^ is better than *M* ^2^ in ***max effectiveness***.

In our verification, we set the *n* of feature set ${S}_{{}_n}^1$ as 10, and compare the ***effectiveness*** of feature spaces from different methods using SVM classifier. For the feature subsets in ${S}_{{}_{10}}^1:{S}_1^1\subset {S}_2^1\subset \dots {S}_9^1\subset {S}_{10}^1$ generated by method *M* ^1^, we compute the classification accuracy on *S*_*k*_^1^ for every *k*(1 ≤ *k* ≤ 10). Then the ***mean effectiveness*** and ***max effectiveness*** of method *M* ^1^ are measured by the average and maximum classification accuracies on ${S}_{{}_{10}}^1$. The results of ***mean effectiveness*** and ***max effectiveness*** on three types of datasets through different methods are shown in Tables 3, 4 & 5 and Additional file 1: Table S1, respectively.

**Table 3:** The results of mean effectiveness on mRNA microarray (top 10)

Methods	SVM-RFE	SVM-RCE	mRMR	IMRelief	SlimPLS	OSFS	FGM	SMKL-FS
Liver	0.913	0.860	**0.965**	0.825	0.831	0.750	0.867	0.963
Pancreatic	0.689	0.777	*0.818*	0.784	0.673	0.707	0.729	0.804
Lung	0.731	0.786	0.942	0.814	0.708	0.704	0.860	*0.964*
Gastric	0.614	0.724	0.688	0.566	0.636	0.533	0.640	*0.760*
Colon	0.736	0.888	0.941	0.803	0.794	0.682	0.812	*0.951*
Breast	0.745	0.776	0.832	0.545	0.693	0.728	0.769	*0.854*
Thyroid	0.835	0.897	0.838	0.633	0.743	0.517	0.802	*0.922*
Prostate	0.577	*0.762*	0.750	0.560	0.682	0.629	0.679	0.717
Mean	0.730	0.809	0.847	0.691	0.720	0.656	0.770	*0.867*

**Table 4:** The results of mean effectiveness on mRNA Sequencing (top 10)

Methods	SVM-RFE	SVM-RCE	mRMR	IMRelief	SlimPLS	OSFS	FGM	SMKL-FS
KIDNEY	0.912	0.952	**0.965**	0.949	0.898	0.914	0.951	0.957
BRCA	0.938	0.982	0.973	0.953	0.871	0.934	0.928	*0.984*
LUNG	0.957	0.977	0.993	0.932	0.942	0.867	0.931	**0.997**
HNSC	0.930	0.949	*0.983*	0.908	0.844	0.900	0.977	0.948
LIHC	0.893	0.937	**0.962**	0.919	0.900	0.798	0.952	0.958
PRAD	0.932	0.928	**0.971**	0.893	0.779	0.764	0.966	0.953
STAD	0.907	0.895	**0.970**	0.945	0.758	0.848	0.898	0.963
THCA	0.945	0.954	**0.975**	0.933	0.883	0.844	0.903	0.970
Mean	0.927	0.947	**0.974**	0.929	0.859	0.859	0.938	0.966

**Table 5:** The results of mean effectiveness on miRNA Sequencing (top 10)

Methods	SVM-RFE	SVM-RCE	mRMR	IMRelief	SlimPLS	OSFS	FGM	SMKL-FS
KIDNEY	0.922	0.832	0.987	0.901	0.896	0.893	0.916	**0.994**
BRCA	0.839	0.963	0.979	0.817	0.973	0.893	0.953	*0.990*
LUNG	0.891	0.946	0.979	0.953	0.831	0.945	0.946	**0.980**
HNSC	0.979	0.955	0.991	0.879	0.874	0.920	0.874	**0.994**
LIHC	0.906	0.836	0.911	0.813	0.871	0.789	**0.925**	0.917
PRAD	0.897	0.933	0.930	0.892	0.905	0.794	0.836	*0.946*
STAD	0.855	0.870	0.853	0.790	0.823	0.760	0.827	*0.880*
THCA	0.925	0.901	**0.969**	0.842	0.876	0.878	0.928	0.967
Mean	0.902	0.904	0.950	0.861	0.881	0.859	0.901	**0.958**

The ***mean effectiveness*** and ***max effectiveness*** of SMKL-FS are better than those from other methods for most datasets of miRNA sequencing, mRNA microarray data and little less than mRMR on mRNA sequencing data. The good performance of mRMR [25] on gene expression data may attribute to the method designed specifically for this type of data. We also see that FGM [33] is the best common method, which has satisfactory performance on different type of gene expression data. The results of accuracy of each *S*_1_^1^, *S*_2_^1^, …, *S*_9_^1^, *S*_10_^1^ on three types of datasets for different methods are shown (See Additional file 2: Figure S1, Additional file 3: Figure S2 and Additional file 4: Figure S3), respectively. In each subgraph, the X-axis represents different feature sets *S*_1_^1^, *S*_2_^1^, …, *S*_9_^1^, *S*_10_^1^, and the Y-axis represents accuracy of each set. For two given feature selection methods *M* ^1^ and *M* ^2^, if the area under the curve of *M* ^1^ is larger than that of *M* ^2^, *M* ^1^ is better than *M* ^2^.

For comparing the performances of the methods using multiple kernels with the method using single kernel, the individual usage and different combination of three kernels, linear kernel *K*(*x*_*i*_, *x*) = (*x*_*i*_, *x*), radial basis function kernel $K\left({x}_i, x\right)= \exp \left(-\frac{{\left\Vert {x}_i- x\right\Vert}^2}{2}\right)$ and polynomial kernel *K*(*x*_*i*_, *x*) = [(*x*_*i*_, *x*) + 1]^2^ are conducted. The results of ***mean effectiveness*** and ***max effectiveness*** on three types of datasets are shown (see Additional file 5: Table S2). In Additional file 5: Table S2, the method using different individual kernels affect the results weakly, and the method using multiple kernels has the best results among the majority of the datasets.

In a practical application, the first step can be skipped. However, because of the existing irrelevant features, when only using the second step, the results are not always better than those after removing the irrelevant features, and meanwhile the process has high computational complexity. Considering the computational complexity, we only test the performance by only using the second step on miRNA datasets. The results are shown in Additional file 6: Table S3. From the table, we can see that the results of only using the second step are not better than those filtering some features in the first step, and meanwhile using all features the second step has high computational complexity.

#### Stability of feature space

The stability of feature space generated from a feature selection algorithm reflects the robustness of the method on different samples of the same type of data [34]. For a list of feature sets $$ {S}_{{}_n}^{11},{S}_{{}_n}^{12},\dots, {S}_{{}_n}^{1 k} $$ generated by method *M* ^1^ on different samples *Ω*_1_, *Ω*_2_, …, *Ω*_*k*_(each *Ω* is a subset of *X*) of dataset *D* and another list of feature sets $$ {S}_{{}_n}^{21},{S}_{{}_n}^{22},\dots, {S}_{{}_n}^{2 k} $$ generated by method *M* ^2^ on samples *Ω*_1_, *Ω*_2_, …, *Ω*_*k*_. Let $$ {I}_1=\left\{{S}_{{}_n}^{11}\cap {S}_{{}_n}^{12}\cap, \dots, \cap {S}_{{}_n}^{1 k}\right\} $$, $$ {U}_1=\left\{{S}_{{}_n}^{11}\cup {S}_{{}_n}^{12}\cup, \dots, \cup {S}_{{}_n}^{1 k}\right\} $$ and $$ {I}_2=\left\{{S}_{{}_n}^{21}\cap {S}_{{}_n}^{22}\cap, \dots, \cap {S}_{{}_n}^{2 k}\right\} $$, $$ {U}_2=\left\{{S}_{{}_n}^{21}\cup {S}_{{}_n}^{22}\cup, \dots, \cup {S}_{{}_n}^{2 k}\right\} $$. If $$ \raisebox{1ex}{$\left|{I}_1\right|$}\!\left/ \!\raisebox{-1ex}{$\left|{U}_1\right|$}\right. $$ is larger than $$ \raisebox{1ex}{$\left|{I}_2\right|$}\!\left/ \!\raisebox{-1ex}{$\left|{U}_2\right|$}\right. $$, we consider the method *M* ^1^ is better than *M* ^2^ in ***union stabilit*** **y** of feature space. For every two samples *Ω*_*i*_, *Ω*_*j*_ ∈ {*Ω*_1_, *Ω*_2_, …, *Ω*_*k*_}, let $$ {R}_{{}_1}^{ij}=\left|{S}_{{}_n}^{1 i}\cap {S}_{{}_n}^{1 j}\right|/\left|{S}_{{}_n}^{1 i}\cup {S}_{{}_n}^{1 j}\right| $$ and $$ {R}_{{}_2}^{ij}=\left|{S}_{{}_n}^{2 i}\cap {S}_{{}_n}^{2 j}\right|/\left|{S}_{{}_n}^{2 i}\cup {S}_{{}_n}^{2 j}\right| $$, if the average of $$ {R}_{{}_1}^{ij} $$ is larger than the average of $$ {R}_{{}_2}^{ij} $$, the method *M* ^1^ is better than *M* ^2^ in ***independent stabilit*** **y** of feature space.

In our verification, we set the *n* of feature sets ${S}_{{}_n}^{11},{S}_{{}_n}^{12},\dots, {S}_{{}_n}^{1 k}$ and feature sets ${S}_{{}_n}^{21},{S}_{{}_n}^{22},\dots, {S}_{{}_n}^{2 k}$ to 100 and use 10-fold cross validation to measure the stability of the feature lists generated by different feature selection methods. Firstly, we randomly choose 90% of the paired samples from each dataset and iterate this process 10 times to obtain 10 different sets for each dataset. Then different feature selection methods are used to select these feature lists. Furthermore, we compute the ***union stabilit*** **y** and ***independent stabilit*** **y** according to the process mentioned above.

**Fig. 1:**
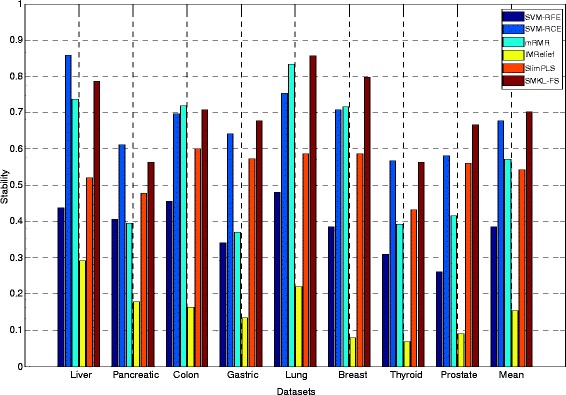
The results of independent stability on different mRNA microarray datasets

**Fig. 2:**
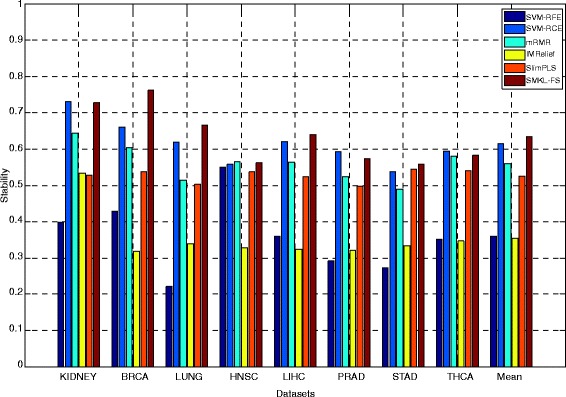
The results of independent stability on different mRNA Sequencing datasets

**Fig. 3:**
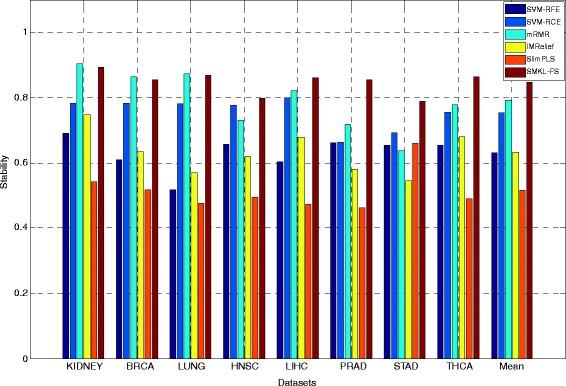
The results of independent stability on different miRNA Sequencing datasets

The results of ***union stabilit*** **y** on three types of datasets through different methods are shown (See Additional file 7: Table S4). From Additional file 7: Table S4, the ***union stabilit*** **y** of SMKL-FS is better than those from other methods on most datasets. The results of ***independent stabilit*** **y** on three types of datasets through different methods are shown in Figs. 1, 2 and 3, respectively. In Figs. 1, 2, 3, the X-axis represents different datasets, and the Y-axis represents ***independent stability***. The ***independent stability*** results of SMKL-FS are better than those from other methods on most datasets.

#### Similarity with other methods

The similarity between the feature space generated from one feature selection algorithm and the feature lists generated by other methods can be used to estimate the availability of the algorithm. For the feature set ${S}_{{}_n}^1$ generated by method *M* ^1^ of dataset *D* and other feature sets ${S}_{{}_n}^2,\dots, {S}_{{}_n}^k$ generated by methods *M* ^2^, *M* ^3^, …, *M* ^*k*^ of the same dataset *D*. Let ${I}_1=\left|{S}_{{}_n}^1\cap {S}_{{}_n}^2\right|$, ${I}_2=\left|\left\{{S}_{{}_n}^1\cap {S}_{{}_n}^3\right\}\right|$,…, ${I}_{k-1}=\left|{S}_{{}_n}^1\cap {S}_{{}_n}^k\right|$, and ${I}_{mean}={\scriptscriptstyle \frac{1}{k-1}}{\displaystyle \sum_{j=1}^{k-1}{I}_j}$. If the *I*_*mean*_ of one method is larger than other methods, the method is better than other methods in ***Similarity***.

In our verification, we set *n* of feature set ${S}_{{}_n}^1$ to 100. Firstly, we select the feature sets ${S}_{{}_n}^1,\dots, {S}_{{}_n}^6$ on each dataset by SVM-RFE, SVM-RCE, mRMR, IMRelief, SlimPLS and SMKL-FS, respectively. Then, for each feature set generated by every method on one dataset, the value *I*_*mean*_ is calculated according to the process mentioned above. The results of ***similarity*** on three types of datasets through different methods are shown in Tables 6, 7 and 8. The ***similarity*** results of SMKL-FS are better than those from other methods on most datasets.

**Table 6:** The results of similarity on mRNA microarray

Methods	SVM-RFE	SVM-RCE	mRMR	IMRelief	SlimPLS	SMKL-FS
Liver	6.33	1.17	**15.83**	1.33	1	15.17
Pancreatic	4.67	0.83	11.17	1.83	3	*16.83*
Lung	3.83	21.83	20.67	0.17	2.17	*23*
Colon	7.17	0.67	19.17	0.67	2.67	*22.67*
Gastric	3.83	0.83	16.00	0.50	3.50	*20.50*
Breast	9.83	32.83	31.83	0	1.67	*33.83*
Thyroid	10.83	29.00	20.17	0	1.67	**29.33**
Prostate	5.50	27.50	20.00	0.50	1.17	*29.17*
Mean	6.50	14.33	19.35	0.63	2.10	*23.81*

**Table 7:** The results of similarity on mRNA Sequencing

Methods	SVM-RFE	SVM-RCE	mRMR	IMRelief	SlimPLS	SMKL-FS
KIDNEY	1.33	8.00	11.00	2.83	1.67	*12.00*
BRCA	5.67	16.83	14.83	3.67	0.83	*17.83*
LUNG	6.50	23.17	11.50	2.83	0.67	*26.67*
HNSC	1.17	*24.17*	11.67	2.50	1.17	23.00
LIHC	9.50	26.67	17.50	1.33	2.33	*29.33*
PRAD	9.83	26.67	19.17	3.33	0.83	*30.00*
STAD	7.83	**29.67**	15.17	16.67	0.33	29.50
THCA	5.17	14.33	12.50	4.83	0.50	*16.00*
Mean	5.88	21.19	14.17	4.75	1.04	*23.04*

**Table 8:** The results of similarity on miRNA Sequencing

Methods	SVM-RFE	SVM-RCE	mRMR	IMRelief	SlimPLS	SMKL-FS
KIDNEY	43.00	33.00	48.50	29.17	28.00	*51.00*
BRCA	39.67	39.33	50.83	25.83	33.00	*52.33*
LUNG	41.50	38.83	50.17	29.50	21.67	*53.33*
HNSC	42.17	38.83	50.50	32.50	22.50	*53.67*
LIHC	42.33	35.50	46.50	24.67	25.17	*47.67*
PRAD	42.33	40.33	53.17	27.00	30.83	*54.33*
STAD	43.50	35.33	48.83	28.67	20.67	*53.33*
THCA	37.33	37.50	47.50	26.50	25.50	*50.83*
Mean	41.48	37.33	49.50	27.98	25.92	*52.06*

## Methods

### Brief review of SVM

Several supervised learning methods, such as Support Vector Machines (SVMs) can be used to analyze data and recognize patterns by classification and regression analysis. The standard SVM algorithm was proposed by Cortes and Vapnik in 1995 [35]. Given a sample set of data points $G={\left\{\left({\overrightarrow{x}}_i,{y}_i\right)\right\}}_{i=1}^n$, ${\overrightarrow{x}}_i\in {R}^m$ and *y*_*i*_ ∈ {+1, − 1}, the decision rule is:

2 $$ \mathrm{S}\mathrm{V}\mathrm{M}(x)=\mathrm{sign}\left({\displaystyle \sum_{i=1}^N{\alpha}_i{y}_i K\left({x}_i, x\right)}+ b\right) $$

where *y*_*i*_ is the class label of the sample *x*_*i*_ and the summation is taken over all the training samples. *α*_*i*_ is the Lagrange multipliers involved in maximizing the margin of separation of the classes. *K*(*x*_*i*_, *x*) is a kernel which can map the feature space to a high dimensional space. There are several popular kernels, such as linear kernel *K*(*x*_*i*_, *x*) = (*x*_*i*_, *x*), radial basis function kernels $K\left({x}_i, x\right)= \exp \left(-\frac{{\left\Vert {x}_i- x\right\Vert}^2}{\sigma}\right)$, homogeneous kernels *K*(*x*_*i*_, *x*) = (*x*_*i*_, *x*)^*d*^ and inhomogeneous polynomial kernels *K*(*x*_*i*_, *x*) = [(*x*_*i*_, *x*) + 1]^*d*^. After obtaining the *α*, we can predict the label of a new data point by the following formula [36]:

3 $$ f(z)={\displaystyle \sum_{i=1}^n{\alpha}_i{y}_i K\left({x}_i, z\right)}+ b $$

and the bias *b* is defined:

4 $$ b=-\frac{1}{2}\left[\underset{\left\{\left. i\right|{y}_i=-1\right\}}{ \max}\left({\displaystyle \sum_{j=1}^n{\alpha}_j{y}_j K\left({x}_i,{x}_j\right)}\right)+\underset{\left\{\left. i\right|{y}_i=+1\right\}}{ \min}\left({\displaystyle \sum_{j=1}^n{\alpha}_j{y}_j K\left({x}_i,{x}_j\right)}\right)\right] $$

### Multiple kernel learning (MKL)

In recent years, several multiple kernel learning (MKL) methods have been proposed to enhance the interpretability of the decision function and improve performances [23, 24]. A convenient approach of MKL is to construct the kernel *K*(*x*_*i*_, *x*) as a convex combination of basis kernels [23]:

5 $$ K\left({x}_i, x\right)={\displaystyle \sum_{m=1}^M{d}_m{K}_m\left({x}_i, x\right)},\kern1.5em \mathrm{with}\kern0.5em {d}_m\ge 0,\kern0.5em {\displaystyle \sum_{m=1}^M{d}_m=1} $$

where *M* is the number of multiple kernels. The kernel *K*_*m*_ may be the popular kernels mentioned above with different parameters. Each single kernel *K*_*m*_ can either use the full set of training samples or subsets of these samples from different data sources. Then, the problem of the model is transferred to the choice of the weights *d*_*m*_.

Actually, the standard primal MKL formulation, which just learns from objective consisting of a simple summation of base kernels subjected to mix-norm regularization, is expressed in a functional form as:

6 $$ \begin{array}{l}\underset{f, b,\xi}{ \min }\ \frac{1}{2}{\left({\displaystyle \sum_m{\left\Vert {f}_m\right\Vert}_{H_m}}\right)}^2+ C{\displaystyle \sum_i{\xi}_i}\hfill \\ {} s. t.\ {y}_i\left({\displaystyle \sum_m{f}_m\left({x}_i\right)}+ b\right)\ge 1-{\xi}_i,\ \forall i\hfill \\ {}{\xi}_i\ge 0\ \forall i\hfill \end{array} $$

where *f*_*m*_ is a function that belongs to corresponding Hilbert space *H*_*m*_, and each Hilbert space *H*_*m*_ endowed an inner product 〈⋅, ⋅ 〉_*m*_ has a unique kernel *K*_*m*_.

However, ${\left\Vert {f}_m\right\Vert}_{H_m}$ is not differentiable. When *f*_*m*_ = 0, it leads to original objective function, which is not smooth. In this article, we apply SimpleMKL [23] that uses a weighted l_2_ norm regularization to calculate the upper bound of the problem through Cauchy-Schwartz inequality. The primal formulation can be replaced as:

7 $$ \begin{array}{l}\underset{f, b,\xi, d}{ \min}\kern0.24em \frac{1}{2}{\displaystyle \sum_m\frac{1}{d_m}{\left\Vert {f}_m\right\Vert}_{H_m}^2}+ C{\displaystyle \sum_i{\xi}_i}\hfill \\ {} s. t.\ {y}_i\left({\displaystyle \sum_m{f}_m\left({x}_i\right)+ b}\right)\ge 1-{\xi}_i,\ \forall i\hfill \\ {}{\xi}_i\ge 0\ \forall i\hfill \\ {}\ {\displaystyle \sum_m{d}_m=1,\ {d}_m\ge 0,\ \forall \mathrm{m}}\hfill \end{array} $$

And the corresponding dual problem is given as follows

8 $$ \begin{array}{l}\underset{\alpha}{ \max }{\displaystyle \sum_i{\alpha}_i}\hbox{-}\ \frac{1}{2}{\displaystyle \sum_{i, j}{\alpha}_i{\alpha}_j{y}_i{y}_j{\displaystyle \sum_m{d}_m{K}_m\left({x}_i,{x}_j\right)}}\hfill \\ {} s. t.\ {\displaystyle \sum_i{\alpha}_i{y}_i=0}\hfill \\ {}0\le {\alpha}_i\le C,\ \forall i\hfill \end{array} $$

where *α* and *C* are Lagrange multipliers of the constrains which related to each data point and their tolerable errors separately.

Note that our new dual objective function is convex and differentiable with respect to α. At each iteration, firstly the coefficients keep unchanged, and the value of objective function is optimized. Then, the coefficients are recovered and updated with above dual variables, and this process repeats until convergence.

### Feature selection algorithm

Similar to other methods [18, 31], our algorithm also tries to construct an efficient process to select a compact set of features. Combined with the multiple kernel learning (MKL) method mentioned in the above section, we present a two-stage feature selection method. For expression data of a set of features, there are four major feature categories: relevant features, redundant features, irrelevant features and noisy features. For two types of expression data, the relevant features are only a very small part. Most of features are irrelevant features, which will be removed firstly by many feature selection methods for expression data analysis. So, in the first stage of our method, the relevant features are identified by measuring score of each feature using the optimizing process of MKL. If the computational complexity is considered, a small set of relevant features in the first step can be selected. In the second stage, an embedded selection scheme, i.e. the forward selection, is applied to search the subset of compact features from the candidate feature sets obtained in the first stage.

#### Selecting the relevant feature set

Firstly, we apply MKL to select the relevant feature set. To implement MKL approach, we select the SimpleMKL method in [23] to obtain the coefficient *d*_*m*_ of the kernel combination . SimpleMKL used an iterative gradient descent process to perform an optimization both on the parameters of the SVM (*α*_*i*_) and the kernel coefficients (*d*_*m*_). There are several kernels can be used, such as linear kernel *K*(*x*_*i*_, *x*) = (*x*_*i*_, *x*), radial basis (RBF) function kernel $K\left({x}_i, x\right)= \exp \left(-\frac{{\left\Vert {x}_i- x\right\Vert}^2}{2{\sigma}^2}\right)$ and polynomial kernels *K*(*x*_*i*_, *x*) = [(*x*_*i*_, *x*) + *c*]^*d*^.

Then the optimal objective function is defined as follows:

9 $$ J=\underset{d_m}{ \min}\underset{\alpha}{ \max } W\left(\alpha, {d}_m\right)\kern0.48em \mathrm{such}\ \mathrm{that}\kern0.62em {\displaystyle \sum_{m=1}^M{d}_m=1}\kern0.5em ,\kern0.36em {d}_m\ge 0 $$

Using SimpleMKL, we can obtain the *J* value for each feature from the total feature set *S* in the process of optimizing *W*(*α*, *d*_*m*_) via $\underset{d_m}{ \min}\underset{\alpha}{ \max } W\left(\alpha, {d}_m\right)$. To select the relevant feature set, the *J* list for features list is computed to measure the relevance between features and samples. Finally, we sort the *J* list in ascend and obtain the ranked features list *S*_*r*_. Then, the top n* features are selected and the feature set ${S}_{n^{*}}$ is obtained. The process of selecting the relevant feature set is defined (See Additional file 8: Table S5).

#### Selecting compact feature subsets

An embedded scheme of the sequential forward selection is utilized to search the compact feature subsets from the relevant feature set ${S}_{n^{*}}$. In general, the wrapper approaches convolve with a classifier (e.g., SVM) and the goals are to minimize the classification error of the dependent classifiers. These wrapper approaches can usually obtain low classification error for their dependent classifiers. However, they have high computational complexity and the selected features are less generalization to classifiers [31]. We use the following formula instead of classification error to carry out the embedded process.

10 $$ {J}_{\mathrm{Z}}=\underset{d_m}{ \min}\underset{\alpha}{ \max}\left({\displaystyle \sum_{i=1}^n{\alpha}_i}-{\scriptscriptstyle \frac{1}{2}}{\displaystyle \sum_{i, j}{\alpha}_i{\alpha}_j}{y}_i{y}_j{\displaystyle \sum_m^M{d}_m{K}_m\left({x}_{{}_i}^{\mathrm{Z}},{x}_j^{\mathrm{Z}}\right)}\kern0.5em \right) $$

where *Z* is the set containing the selected features, such as *Z* = {*f*_1_, *f*_2_, …, *f*_*n*_}. In this article, the *J*_Z_ is calculated by using SimpleMKL method [23], which solves the MKL problem through a primitive formulation involving a weighted l2-norm regularization [23].

Then, a forward process is used to to select the subset with *r* features from ${S}_{n^{*}}$ by the incremental manner. And initially, the score of *J*_0_ is set to + ∞ and the subset *Z* is set to empty. We search each feature in the feature subset, such as *f*_1_, *f*_2_, …, *f*_*n*_, and compute the objective functions ${J}_{f_1},{J}_{f_2},\dots, {J}_{f_n}$ using SimpleMKL. The feature *f*_i_ which generates the largest $\varDelta J={J}_0-{J}_{f_{\mathrm{i}}}$ reduction is appended to *Z*. Then, the algorithm selects the feature *f*_j_ which generates the largest *ΔJ* reduction from the set $\left\{{S}_{n^{*}}- Z\right\}$ to *Z*. The process of incremental selection will repeat until *ΔJ* ≤ 0 or the given iterations. The process of selecting compact feature subsets is defined (See Additional file 8: Table S6).

## Discussion and conclusions

With the development of high-throughput microarray chip and RNA sequencing technology, we can obtain a large number of expression data with different types. The researchers can acquire these data from several public databases, such as GEO, SMD, ArrayExpress and TCGA. However, because the transcriptomics experiments have high cost, most of these data have samples with small size and tens thousands genes or hundreds miRNAs. How to extract informative features from expression data effectively and robustly is a challenging and crucial problem for expression data analysis. Feature selection technique had been widely applied to select a subset of relevant features and eliminate redundant, irrelevant and noisy features.

In general, most feature selection methods can be divided into three categories: filter, wrapper and embedded. Filter methods independent of the classifier, select relevant features only relying on the intrinsic properties of expression data. Filter methods contain two subclasses: univariate and multivariate. Univariate methods are processed by filtering single feature and multivariate methods are used to select features by considering combination of features. The advantages of univariate methods are fast, scalable and independent of the classifier, and the disadvantages of these methods are thoughtlessness of feature dependencies and ignoring the interaction with the classifier. The advantages of multivariate methods contain: feature dependencies, independent of the classifier and better computational complexity than wrapper methods. But the multivariate methods are slower and less scalable than univariate methods. Wrapper approaches, which can be divided into deterministic and randomized types, generate the scores for features and select them based on the classifier. The deterministic methods, which are simple, have less computational complexity and more risk of over fitting than randomized methods. But they are more prone to get a result of local optimum than randomized methods. Embedded approaches, which have lower computational complexity than wrapper methods, select optimal feature subset based on classifier construction in the combined space of feature subsets and hypotheses.

Most of above methods construct the feature selection model on individual expression data simply, and they rarely consider the effectiveness and stability on expression data from different type of expression data. In order to overcome the disadvantages of above methods, a hybrid feature selection method based on multiple kernel learning is proposed. We evaluate performance of method on expression dataset of different types. Except for comparing the classification accuracy with other methods, we also compare the performances of different algorithms through measuring the stability, similarity and consistency. The experimental results show that the proposed method has a satisfactory capability of feature selection for different expression datasets analysis.

The kernel methods and other machine learning methods always have the problem of overfitting, especially in small sample size. And, one of characteristics of gene expression data is high dimensional and small sample size. There are commonly used methodologies to avoid overfitting on machine learning: Regularization, Cross-Validation, Early Stopping and Pruning. The regularization part adds a cost term for bringing in more features with the objective function. Hence, regularization can make the coefficients for many variables to zero and hence avoid the overfitting. Cross validation can identify the methods, which may have poor performance generating by overfitting training on several datasets. The methods of early stopping try to prevent overfitting by controlling the number of iterations on iterative method. Pruning methods, which remove the nodes with little predictive power, are used for several methods based on tree. In this article, we used regularization and sequential forward selection method to decrease the influence of overfitting on small sample size. With the lower price of Mircoarray and RNA sequencing, the samples are more and more obtained from individual experiment, such as the new experiment of RNA sequencing on single-cell, which can handle more than 4000 samples [37]. So, in the future, the influence of overfitting on expression data analysis will be getting smaller and smaller, and machine learning methods and kernel methods will be better used with these data.

## Additional files

Additional file 1: Table S1. The results of max effective on mRNA microarray, mRNASeq and miRNASeq datasets. (XLSX 11 kb)
Additional file 2: Figure S1. Classification accuracy of features combination on different mRNA microarray datasets. (PDF 77 kb)
Additional file 3: Figure S2. Classification accuracy of features combination on different mRNASeq datasets. (PDF 61 kb)
Additional file 4: Figure S3. Classification accuracy of features combination on different miRNASeq datasets. (PDF 52 kb)
Additional file 5: Table S2. The results of mean and max effective by different kernel. (XLSX 12 kb)
Additional file 6: Table S3. The results of mean and max effective by using step one. (XLSX 8 kb)
Additional file 7: Table S4. The results of union stability. (XLSX 11 kb)
Additional file 8: Tables S5 and S6. The pseudo code of proposed algorithm. (DOCX 50 kb)

## Abbreviations

GEO: Gene Expression Omnibus
miRNA: microRNA
MKL: Multiple kernel learning
MRMR: Minimum Redundancy Maximum Relevancy
mRNA: messenger RNA
RFE: Recursive Feature Extraction
RNA: Ribonucleic acid
SMD: Stanford Microarray Database
SMKL-FS: Simple MKL-Feature Selection
SVM: Support Vector Machine
SVM-RFE: Support Vector Machine-Recursive Feature Elimination
TCGA: The Cancer Genome Atlas
