# Supplementary figures and images for: A feature selection method based on multiple kernel learning with expression profiles of different types
Source: BioData Min. 2017 Feb 2;10:4. doi: 10.1186/s13040-017-0124-x (PMC5288949; doi:10.1186/s13040-017-0124-x)

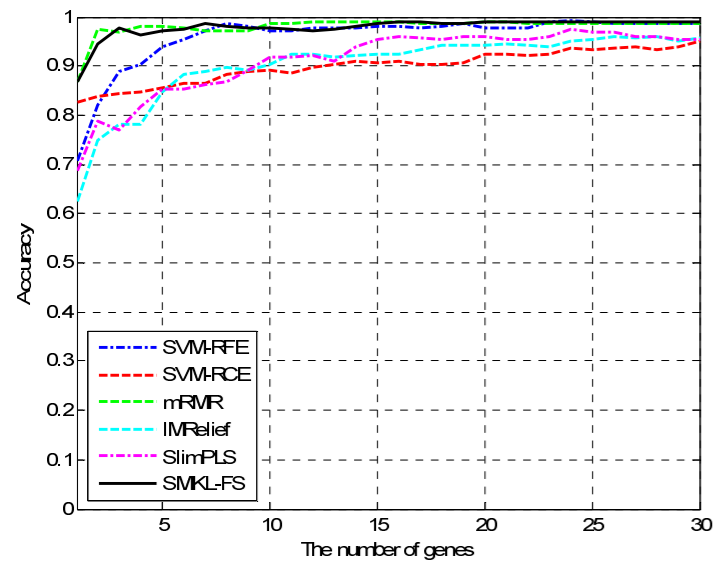

Liver

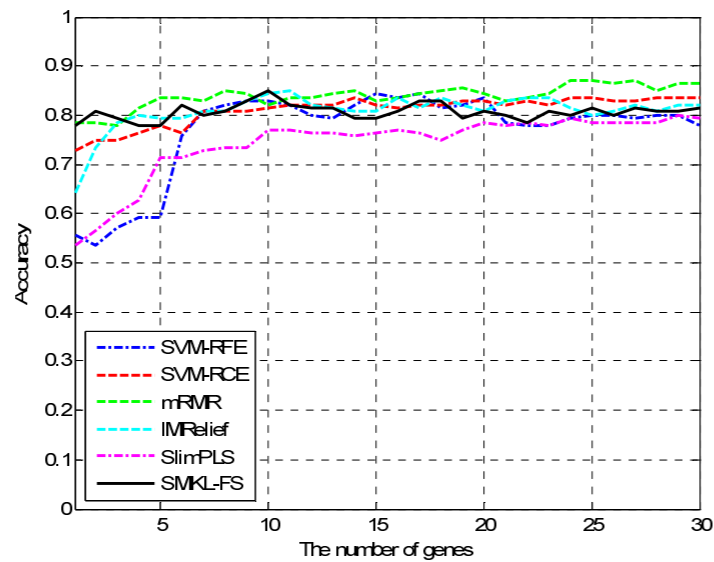

Pancreatic

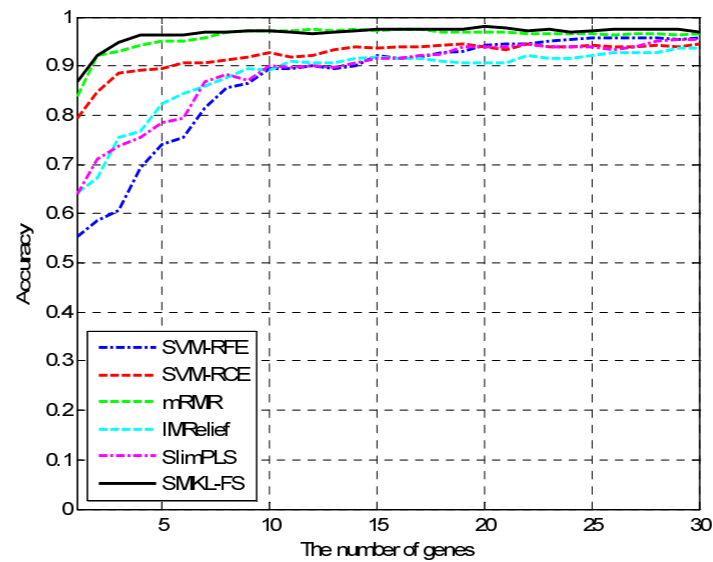

Lung

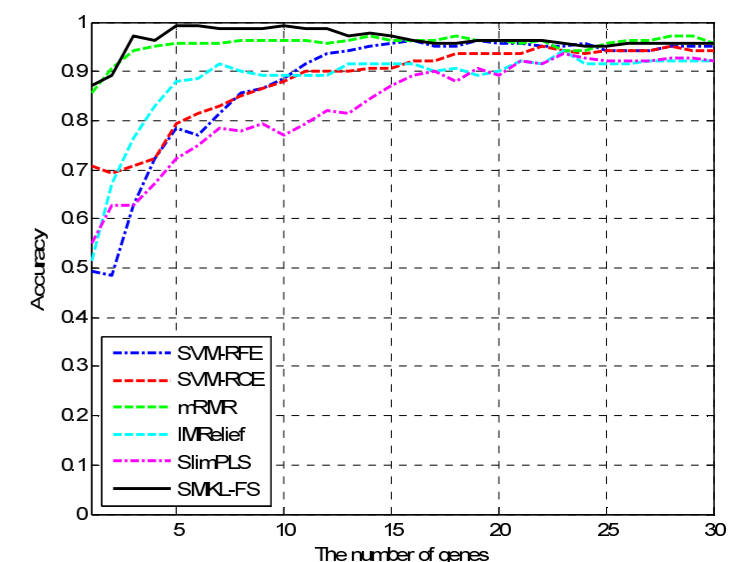

Colon

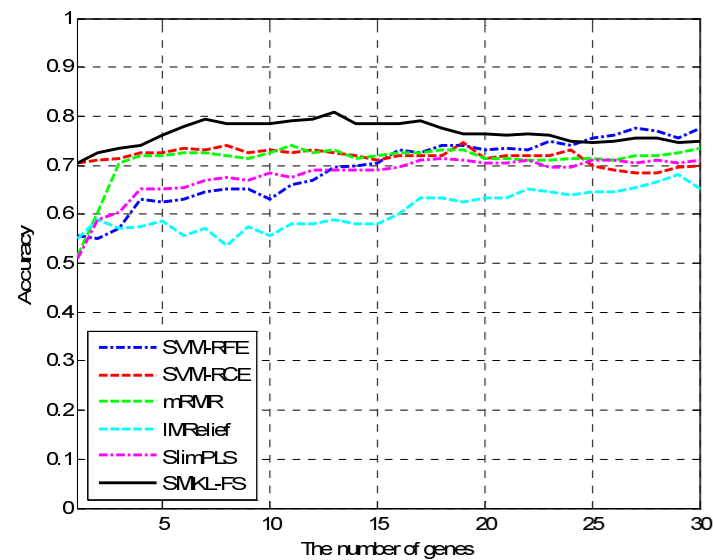

Gastric

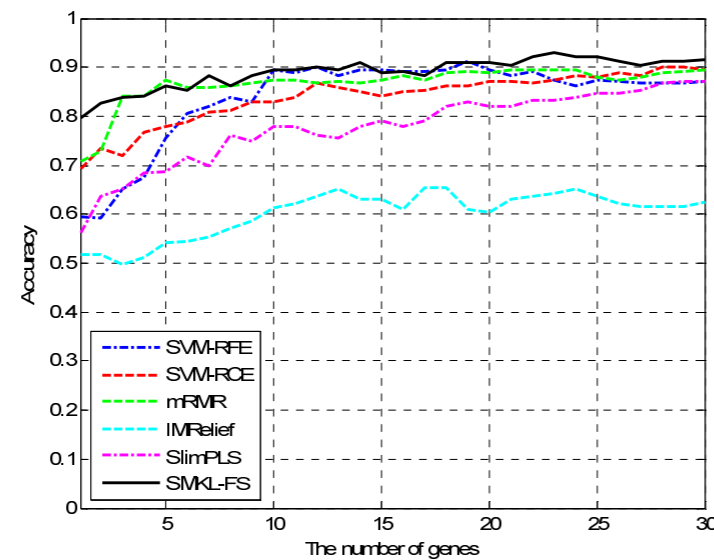

Breast

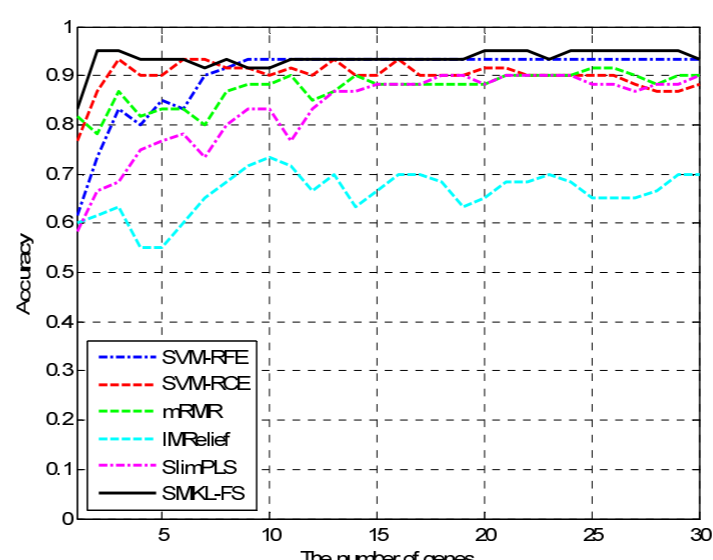

Thyroid

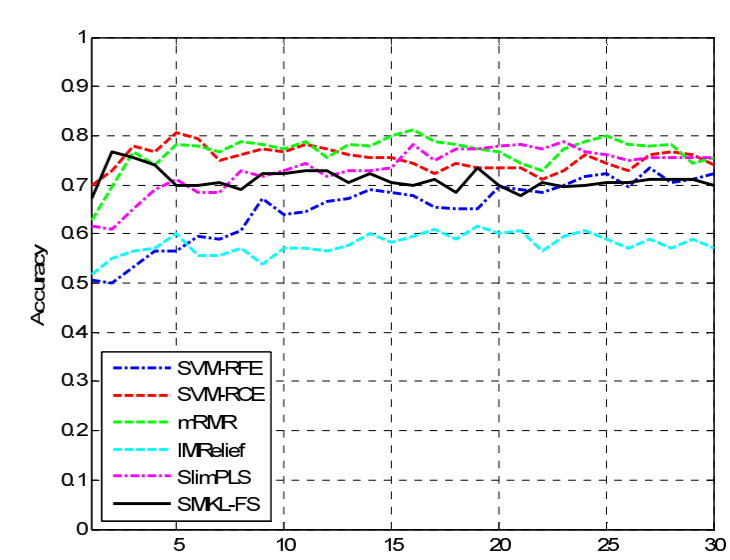

Prostate

Supplement: Additional file 2: Figure S1. — Classification accuracy of features combination on different mRNA microarray datasets. (PDF 77 kb) [file 13040_2017_124_MOESM2_ESM.pdf]

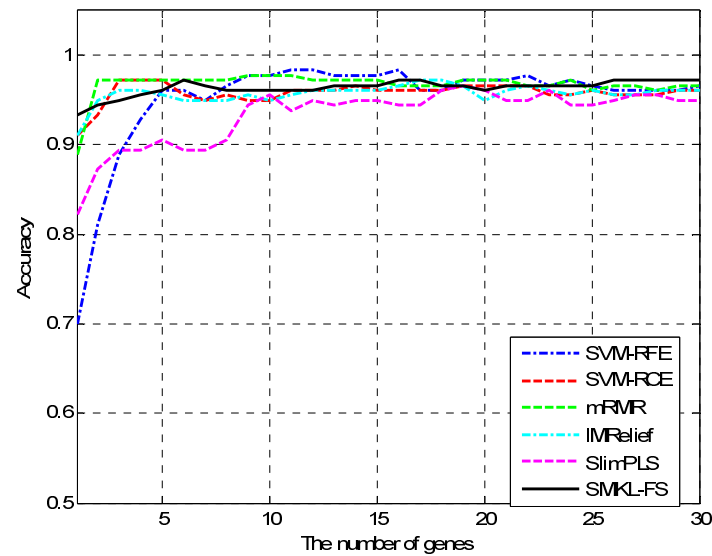

KIDNEY

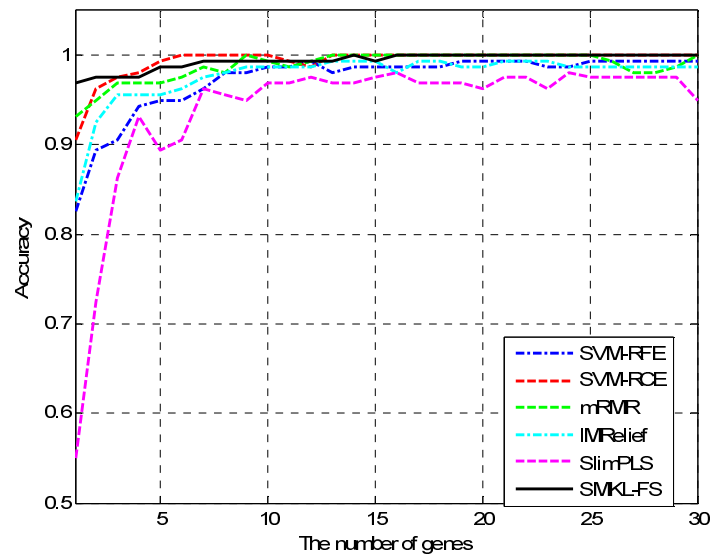

BRCA

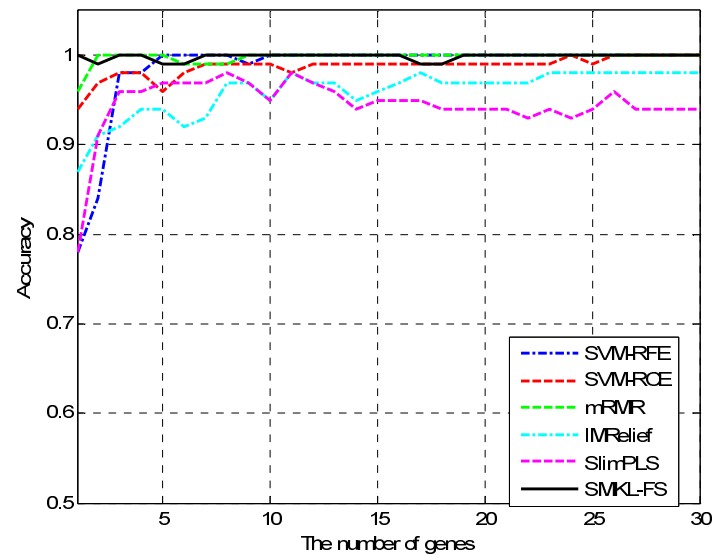

LUNG

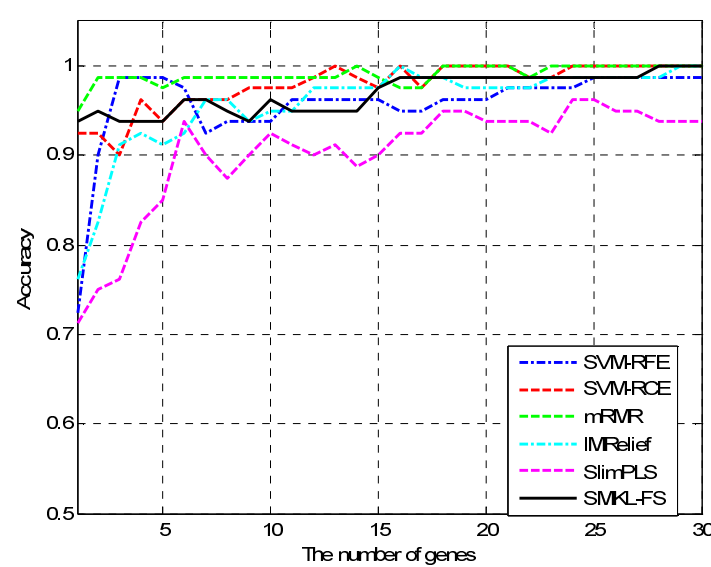

HNSC

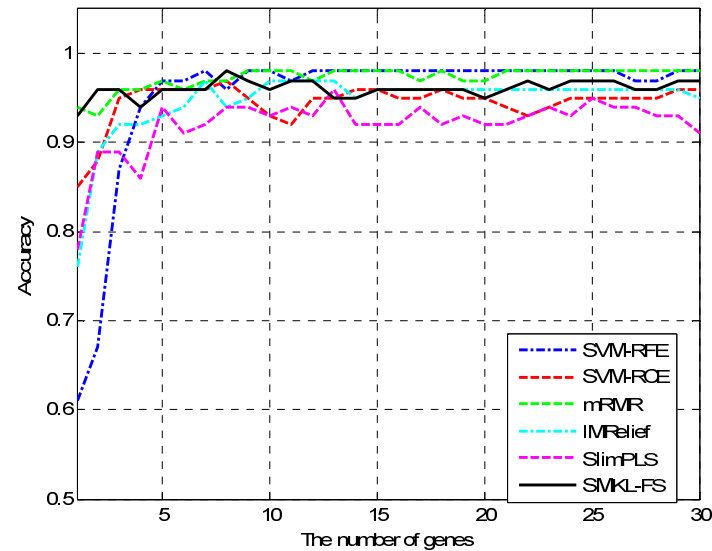

LIHC

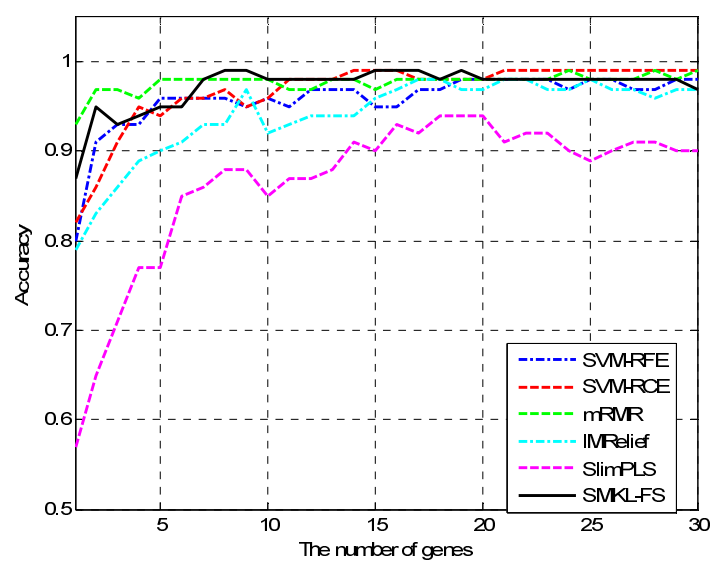

PRAD

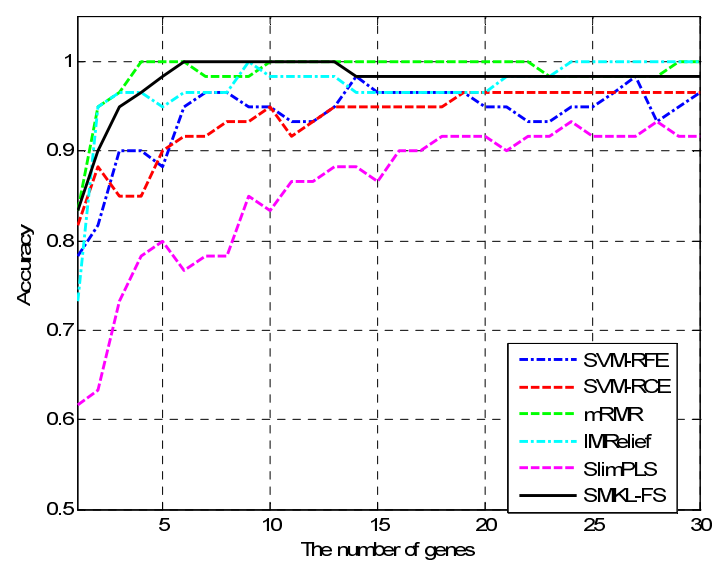

STAD

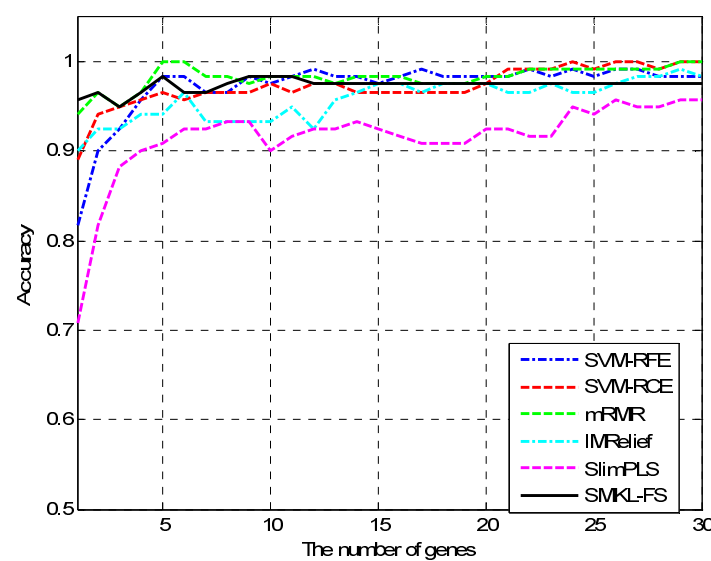

THCA

Supplement: Additional file 3: Figure S2. — Classification accuracy of features combination on different mRNASeq datasets. (PDF 61 kb) [file 13040_2017_124_MOESM3_ESM.pdf]

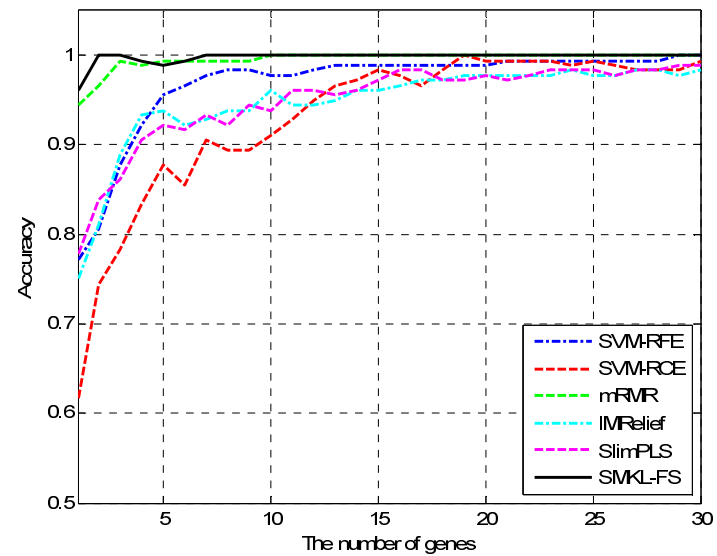

KIDNEY

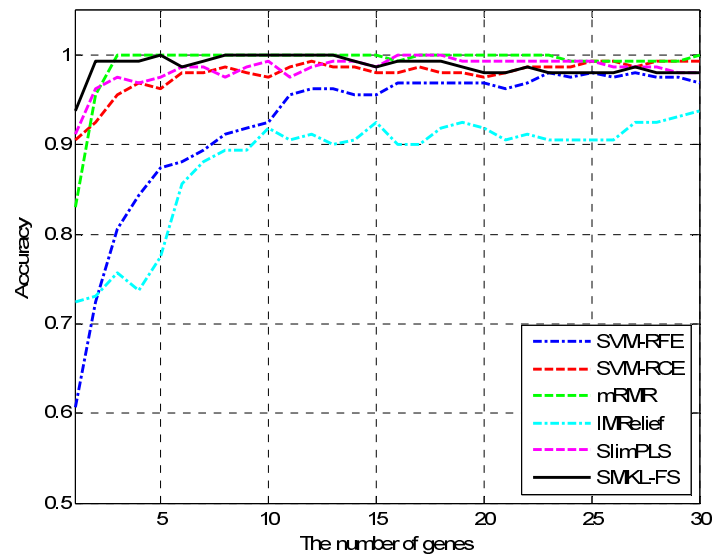

BRCA

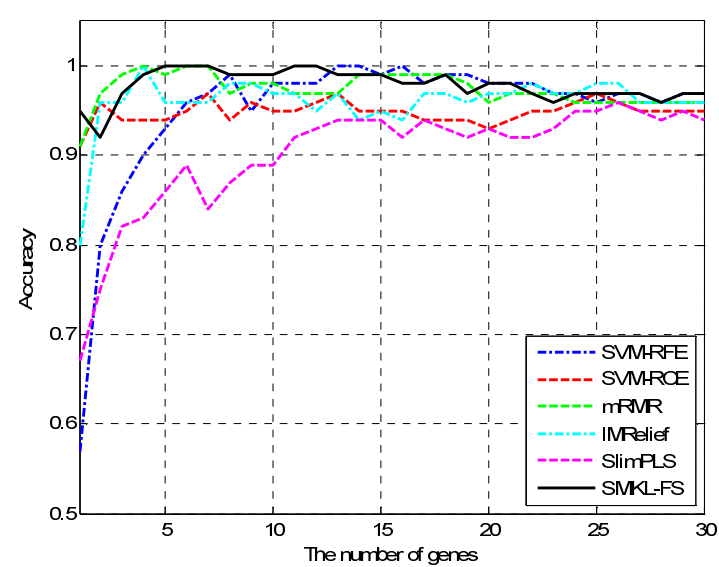

LUNG

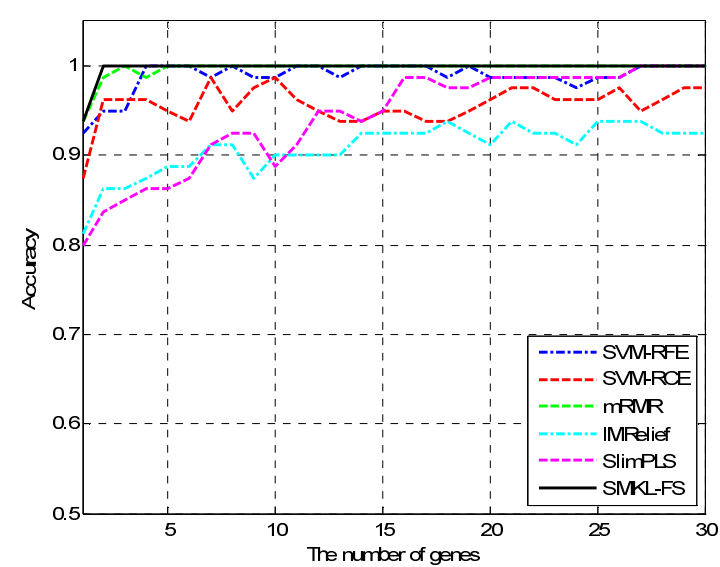

HNSC

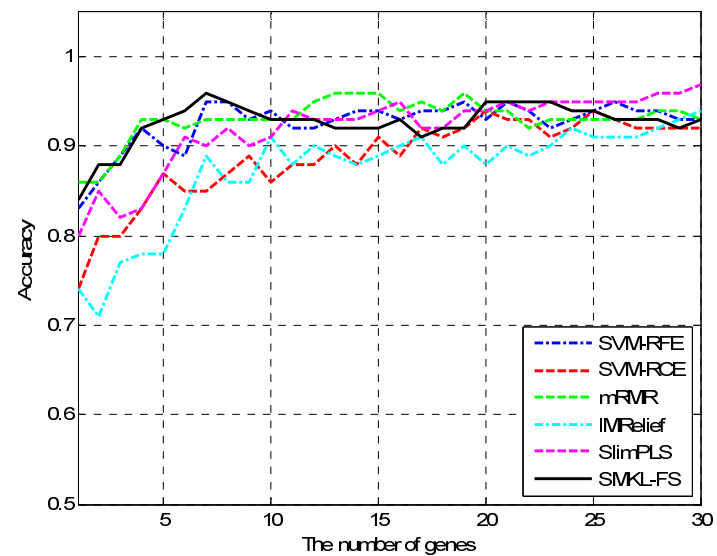

LIHC

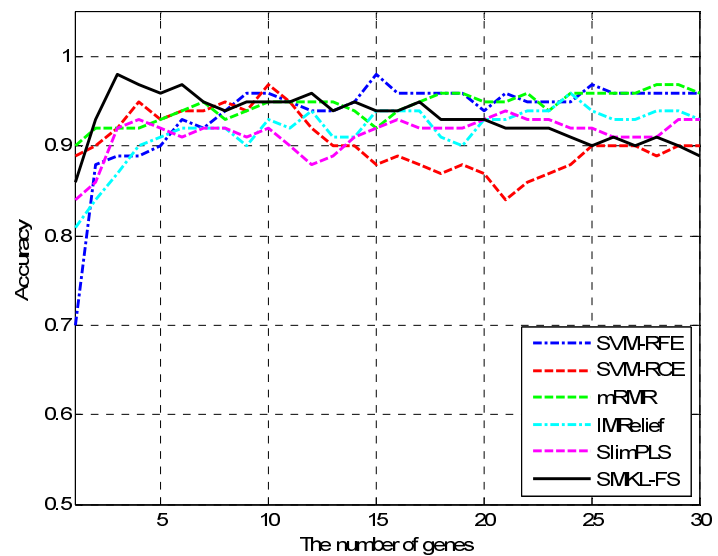

PRAD

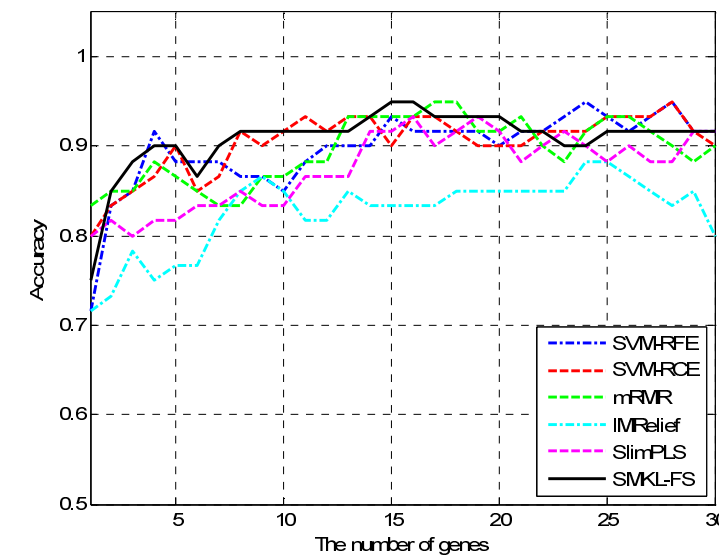

STAD

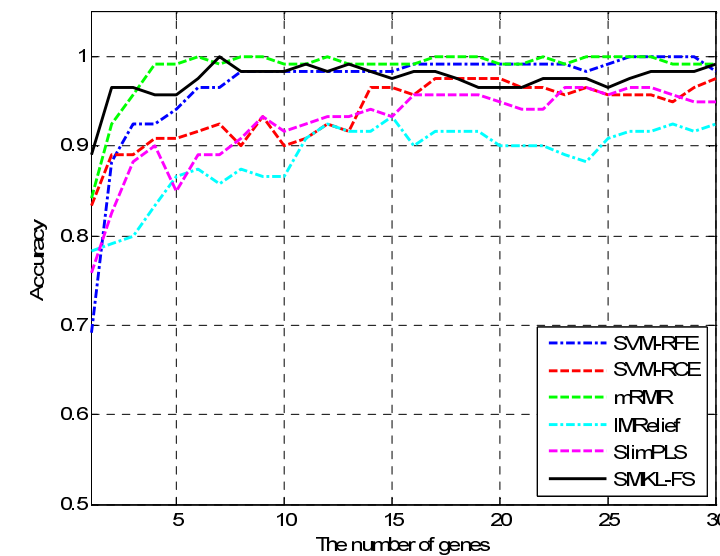

THCA

Supplement: Additional file 4: Figure S3. — Classification accuracy of features combination on different miRNASeq datasets. (PDF 52 kb) [file 13040_2017_124_MOESM4_ESM.pdf]
