# Supplementary material for: A feature selection method based on multiple kernel learning with expression profiles of different types
Source: BioData Min. 2017 Feb 2;10:4. doi: 10.1186/s13040-017-0124-x (PMC5288949; doi:10.1186/s13040-017-0124-x)
Supplement: Additional file 8: Tables S5 and S6. — The pseudo code of proposed algorithm. (DOCX 50 kb) [file 13040_2017_124_MOESM8_ESM.docx]

| **Selecting the Relevant Feature Algorithm：** |
| --- |
| **Input:**  Training examples: $\text{X}_{0}=[x_{1},x_{2},... x_{i},... x_{N}]^{T}$  Class label: $\text{y}=[y_{1},y_{2},... y_{i},... y_{N}]^{T}$  List of features: $S=[1,2,... ,f]$  Relevant feature number: $n^{*}$  **Initialize:**  **for** each feature *f* of *S* **do**  compute list of $J$ by using SimpleMKL  **end for**  sort $J$ ascend and get the ranked features list $S_{r}$  select top $n^{*}$ features, obtained feature set $S_{n^{*}}$  **Output:**  Feature set $S_{n^{*}}$ |
|  |

Table S3: The pseudo code of the Relevant Feature Selecting Algorithm

Table S4: The pseudo code of the Selecting Compact Feature Subset Algorithm

| **Selecting Compact Feature Subset** **Algorithm：** |
| --- |
| **Input:**  Training examples: $\text{X}_{0}=[x_{1},x_{2},... x_{i},... x_{N}]^{T}$  Class label: $\text{y}=[y_{1},y_{2},... y_{i},... y_{N}]^{T}$  Relevant Feature Set: $S_{n^{*}}$  **Initialize:**  Feature Subset: $Z=[]$  Set score of $J_{0}=+\infty$  **while** $\Delta J\leq0$ or $\text{length}\left(Z\right)\leq k$ **do**  compute $J_{Z}=\min_{d_{m}} \max_{\alpha} W(\alpha,d_{m})$ of selected features set *Z* by using SimpleMKL  **for** each feature $i\in\{S_{n^{*}}-Z\}$ **do**  compute $J_{Z+i}$ of selected features set $\{Z,i\}$ by using SimpleMKL  compute $\Delta J_{i}=J_{Z}-J_{Z+i}$  **end for**  select feature *f* which generates the largest $\Delta J$ reduction and $Z=\{Z,f\}$  **end while**  **Output:**  Feature ranked list *Z*. |
